# SNPL: One Scheme of Securing Nodes in IoT Perception Layer

**DOI:** 10.3390/s20041090

**Published:** 2020-02-17

**Authors:** Yongkai Fan, Guanqun Zhao, Kuan-Ching Li, Bin Zhang, Gang Tan, Xiaofeng Sun, Fanglue Xia

**Affiliations:** 1 Institute of Computer Science and Cybersecurity, Communication University of China, Beijing 100024, China; fanyongkai@gmail.com; 2 Tus College of Digit, Beijing 100084, China; 3 Department of Computer Science and Technology, China University of Petroleum, Beijing 102249, China; 2017011316@student.cup.edu.cn (X.S.); 2017011320@student.cup.edu.cn (F.X.); 4 Department of Computer Science and Information Engineering, Providence University, Taichung 43301, Taiwan; 5 Department of Electrical Engineering, University of South Carolina, Columbia, SC 29208, USA; zhangbin@cec.sc.edu; 6 Department of Computer Science and Engineering, Penn State University, University Park, PA 16802, USA; gtan@cse.psu.edu

**Keywords:** IoT, security, security framework, IoT nodes

## Abstract

The trustworthiness of data is vital data analysis in the age of big data. In cyber-physical systems, most data is collected by sensors. With the increase of sensors as Internet of Things (IoT) nodes in the network, the security risk of data tampering, unauthorized access, false identify, and others are overgrowing because of vulnerable nodes, which leads to the great economic and social loss. This paper proposes a security scheme, Securing Nodes in IoT Perception Layer (SNPL), for protecting nodes in the perception layer. The SNPL is constructed by novel lightweight algorithms to ensure security and satisfy performance requirements, as well as safety technologies to provide security isolation for sensitive operations. A series of experiments with different types and numbers of nodes are presented. Experimental results and performance analysis show that SNPL is efficient and effective at protecting IoT from faulty or malicious nodes. Some potential practical application scenarios are also discussed to motivate the implementation of the proposed scheme in the real world.

## 1. Introduction

The Internet of Things (IoT) is well-known for the integration of several technologies with communication systems [1]. The prosperity of IoT is not the reason to neglect its security issues. In fact, the security of IoT is far worse than people know in this respect. There are numerous examples in the real-world about IoT security. For instance, the embedded Radio Frequency Identification (RFID) tags in devices and equipment can transmit or reply to messages [2]. Without appropriate authentication mechanisms, data that are collected by sensor networks may be accessed or distorted by attackers [3]. The sensor system works in unattended status, such that adversaries can modify the information stored in the nodes or decides when the data are delivered to the destination [4]. In the case of node capture attacks, adversaries can capture or control smart devices, by physically replacing or tampering with the nodes and disguising a malicious node like a normal node, to interface with the system [5,6]. In these attacks, malicious nodes can transfer legal identity information, which was received from normal nodes to the target hosts, so that the rogue devices gain trust in IoT networks [7]. Moreover, attackers can crack and gain the encryption key by using cryptanalysis attacks [8] or timing attacks [9]. Notably, this means that side-channel attacks can be used to illegally change or control intelligent devices such as IoT nodes [8].

To mitigate security risks, several security solutions have been proposed in recent years. Babar et al. [9] proposed an embedded security scheme to strengthen the internal security of a device itself by prevention, diagnosis, and elimination. Pacheco et al. presented a security framework that aims at smart cyberinfrastructures [10]. Besides, they proposed a general threat model that can be used for exploiting new safeguard methodology for IoT devices averting known or unknown attacks. Huang et al. developed a prototype security framework called SecIoT that provides a transparent and robust protection mechanism for relieving security threads [11]. Kalra et al. presented a protocol framework for mutual authentication based on Elliptic Curve Cryptography (ECC), which aims to achieve secure communication between embedded devices and the cloud [12]. Zhou et al. proposed a novel scheme of media awareness to promote the security heterogeneity of multimedia applications within a sensor network [13]. Tao et al. presented a framework of security services based on ontology, for the sake of ensuring confidentiality during an interactive process under an intelligent residential environment [14]. Kang et al. proposed a security framework and combined it with self-signature and access control technologies to avoid attacks and ensure the security of a smart home environment [15]. Meidan et al. used machine learning algorithms to identify and classify IoT devices via network traffic data, and proposed a scheme [16].

**Figure 1:**
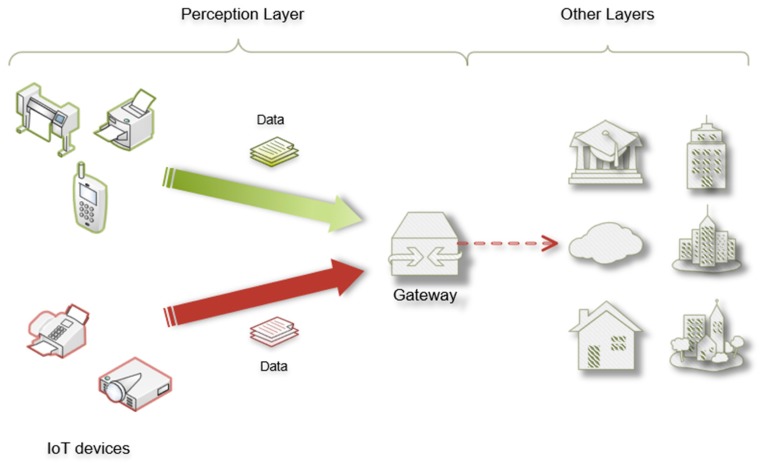
The attacker model in the real scenario.

With these reported works, however the security of IoT nodes was not thoroughly studied, which leads to weakness in the security of the perception layer. The perception layer is the bottom of IoT, containing various IoT devices for collecting or measuring data from the real physical world, and then transferring to upper layers [17]. It can be said that this layer is of vital importance as it controls the source of the data, playing the role of “the last mile of communications” in IoT. Moreover, IoT nodes are the source of data, which is critical to the security of IoT. To address this problem, this paper develops a Securing Node in Perception Layer (SNPL) scheme. As shown in Figure 1, the primary purpose of the SNPL scheme is to ensure data authenticity from sources. In IoT nodes, the most important ones are sensors as they collect and transfer data for further use. The authenticity and trustworthiness of IoT nodes are critical for data analysis. The gathered data is useless if it is not from true nodes. The worst part of the entire thing is that data from malicious nodes, which are changed on purpose, can cause catastrophic consequences for decision-making systems based on them. By realizing this, SNPL aims to distinguish authentic nodes from malicious nodes to guarantee the reliability of collected information. To achieve this goal, a security scheme is proposed to ensure the trustworthiness of nodes in IoT perception layers. Different from other schemes [9,10,11,12,13,14,15,16], all sensitive operations are implemented in the Trusted Execution Environment (TEE), while general operations are carried out in the Rich Execution Environment (REE). TEE protects susceptible operations during the detection of malicious nodes, which enables isolated execution for key-related operations, access policy design, and identity recognition. Unique values and improved attribute-based signatures are used for the identification of IoT nodes.

The main contributions of this paper are outlined, as the following:

1. Propose a security scheme for the perception layer of IoT to identify and avoid potential hazards caused by unsafe nodes,
2. Combine the TEE technology with the SNPL scheme to provide a safe isolation space for sensitive operations, such as key generation and node identification,
3. The scheme satisfies the requirement of Confidentiality, Integrity, and Availability (CIA), as well as the lightweight property, which offers higher availability and portability for application in IoT devices, and
4. Use unique information of each device node as a kind of identifier such that forgery attacks and substitution attacks can be effectively reduced. Furthermore, appropriate encryption technologies can be integrated to enhance the security of the algorithm.

Note that this paper mainly focuses on the effectiveness and accuracy of the proposed framework based on our algorithm, of which the results are expressed as the success rate. Besides, the performance tests in experiments pay close attention to the various types and quantities of nodes in a small-scale local IoT. The later experiments are designed and simulated with the Raspberry Pi instead of real-time applications on a hardware device, for the processing time aiming at highly time-sensitive applications is not our research focus. Meanwhile, the performance of transmitting periodically nodes, sensors transmitting at fixed periodic time slots, or other conditions are not within the scope of our research either.

The remainder of this paper is organized, as follows. Section 2 introduces technical preliminaries such as TEE, Attribute-Based Signature (ABS) scheme, and a brief introduction of number theory, which are the technical base of the proposed scheme. Section 3 describes the SNPL scheme in detail. Section 4 presents experiments and evaluation metrics to assess the performance of SNPL. Section 5 discusses the possible applications, which is followed by concluding remarks in Section 6.

## 2. Preliminaries

This section briefly introduces key technologies used in the proposed scheme. These technologies include TEE, ABS scheme, and two mathematical concepts, which are grouped with bilinear pairings and monotone span programs. 

### 2.1. Trusted Execution Environment (TEE)

TEE is a tamper-resistant processing environment, which is independent of the normal environment [18]. It runs on an isolated kernel and has the ability to fight against physical attacks as well as software attacks in the main memory. The substance of TEE is dynamic and updated securely [19]. Usually, TEE is used to execute sensitive operations such as encryption or key generation, and it often has more restricted functions and rooms than REE.

**Figure 2:**
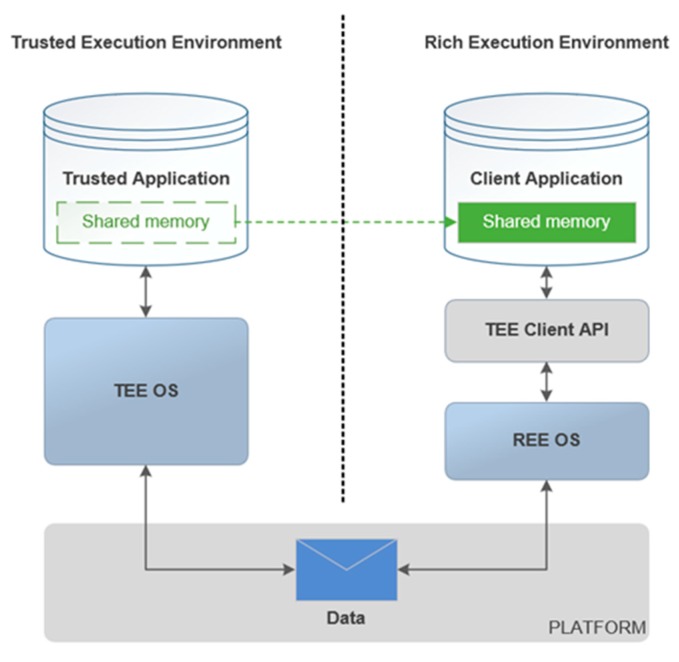
The interaction between Trusted Execution Environment (TEE) and Rich Execution Environment (REE).

Briefly, TEE is constructed to run security services, while REE is a platform for devices to request services. REE represents a normal processing environment with rich functions. For example, Windows, Linux, Android, and IOS can be referred. The basic interaction process between TEE and REE is supported by client Application Programming Interface (API) and shared memory as shown in Figure 2. This interaction process provides a safe and feasible way to transfer information between the two isolated execution environments.

### 2.2. Attribute-Based Signature (ABS) Scheme

The ABS scheme is a multifunctional control scheme. It allows for users to sign a message by taking advantage of fine-grained controls over identifying information. For this, the identity of a signer is uniquely represented by a collection of attributes and a signature of the signer is generated based on these attributes [20]. Afterward, the signature result can be used, for instance, for verifying a user’s identification. In short, the rights of users depend on their attributes. Besides, more application samples can be referred in [20].

A typical ABS scheme has four main steps, which are presented [21]:

1. Setup: The authority or trusted third-party acquires a key pair: Public key (*PK*) and Master key (*MK*). Then, the *PK* will be opened and the *MK* will be kept privately. Both *PK* and *MK* are generated by a series of parameters (denoted as *para*). This step is shown as (*PK*, *MK*) ← *Setup* (*para*).
2. KeyGen: In order to assign users a set of attributes (denoted as *Attr*), the third party or authority generates a Signing key (*SK*). The *SK* is given to users for further use. This step is expressed as *SK* ← *KeyGen*(*PK*, *MK*, *Attr*).
3. Sign: In this step, the user obtains a Signature *σ* on the basis of a Claim-Predicate *Υ*, along with the *PK*, *SK* and the attribute set. The user can then use *σ* to sign a message *m*. The process is represented as *σ* ← *Sign*(*PK*, *SK*, *m*, *Υ*).
4. Verify: To verify the Signature of the message with the Predicate *Y*, this step employs a Boolean function *value* ← Verify(*PK*, *m*, *Υ*, *σ*). According to the output, target parties can judge the identity of data generators.

### 2.3. Groups with Bilinear Pairings

Let $G_{1}$, $G_{2}$ and $G_{T}$ be the cyclic multiplicative groups, whose orders are all a prime p. Let $g_{1}$ and $g_{2}$ be the generators of $G_{1}$ and $G_{2}$ separately, and a map $m:G_{1}\times G_{2}\to G_{T}$. If $m\left(g_{1},g_{2}\right)$ is a generator of $G_{T}$ then $m:G_{1}\times G_{2}\to G_{T}$ is a bilinear pairing and it has the following properties [22]:

1. $m\left(g_{1}{}^{a},g_{2}{}^{b}\right)=m{\left(g_{1},g_{2}\right)}^{ab}$;
2. There exists $g_{1}\in G_{1},g_{2}\in G_{2}$ that satisfy $m\left(g_{1},g_{2}\right)\neq 1$;
3. There is always an effective method to calculate $m\left(g_{1},g_{2}\right)$ for all $g_{1}\in G_{1},g_{2}\in G_{2}$.

### 2.4. Monotone Span Programs

Suppose there is a matrix M with *l* rows and *t* columns, and a nonzero row vector $\vec{v}$, of which the number of coordinates is identical with the number of columns in M. A span program over a field F is expressed as $S=\left(M,\mu ,\vec{v}\right)$, in which M is the matrix with entries of F, $\vec{v}$ is a target vector, and μ is the labeling of the rows of M. 

A monotone span program means the labels of rows are simply positive literals $\left\{x_{1},\ldots x_{n}\right\}$. The calculation results of monotone span programs are only monotone functions, and one monotone span program can calculate a monotone Boolean function [23]. 

Suppose δ is a monotone Boolean function. A monotone span program for δ over a domain D is a matrix $M_{l\times t}$ with entries in D. Besides, it includes a labeling function μ related to rows of M with input variables of δ. The relationship between δ and M is as follows: $\delta \left(x_{1},x_{2},\ldots ,x_{n}\right)=1$ if and only if $\vec{v}M=\left[1,0,0\ldots ,0\right]$.

## 3. The SNPL Scheme

In this section, the SNPL scheme is elaborated for the improvement of reliability and robustness. First, the node fingermark concept is described to ensure the uniqueness of the node. By doing so, the SNPL scheme can separate the true node from others. Second, the design of the scheme is discussed. Finally, the security proof of the proposed design is provided.

### 3.1. Node Fingermark

To exploit the unique identification information of an IoT node, the concept of fingermark is used to certify objects by unique features extracted from equipment information. Aside from the Universally Unique Identifier (UUID) of a device, more complex information or node attributes can be included. In the SNPL scheme, the Unique Identifier of a hardware device, which is the unique hardware configuration information of an IoT node, is the original information. If the node is replaced by a new one, the information will change correspondingly. The hash algorithm is used for a hashing of the information. The result of encryption is considered as a fingermark value of a device node. The result of hash value can be used to ensure the trustworthiness of data sources and, therefore, the identity of the node is guaranteed.

### 3.2. Scheme Design

**Figure 3:**
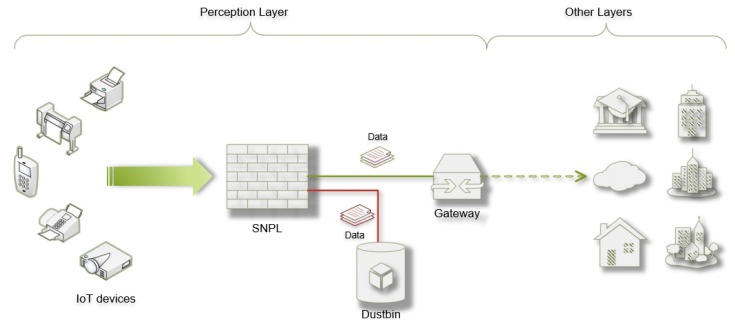
The usage scenario of SNPL in the IoT environment.

Figure 3 shows a usage scenario of the SNPL scheme in the IoT environment. Suppose there is an IoT network made up of many IoT nodes. Device nodes are located throughout the physical world and they gather information by various kinds of sensors embedded inside. The collected information will then be transmitted to the key nodes (for example, the gateways in Figure 3) in IoT. Finally, the key nodes transfer the processed data to other consumers. The main uncertainty for the security of the process is that the originality of data, such as the data gathered in key nodes, cannot be guaranteed. This is a hidden danger at the beginning of the process and may lead to failure of the whole process. To solve this problem, a security scheme is injected into the perception layer of IoT, between endpoint nodes (i.e., the IoT devices in Figure 3) and key nodes. The security scheme can effectively reduce hazards from the beginning by distinguishing nodes with the opposite status.

**Figure 4:**
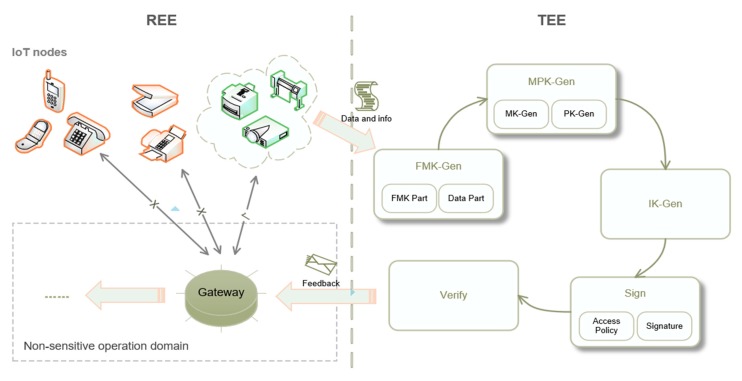
The dominant modules in a secure scheme.

Figure 4 exhibits the dominant modules in the proposed security scheme, which is divided into two parts of execution by employing the REE and TEE technologies. Both parts are running on the same device as well as the object to build the SPNL scheme. In this scheme, REE runs common insensitive operations and connects unknown nodes. TEE runs the following five modules as trusted applications: *i*. *FMK-Gen* (responsible for fingermark generation of devices); *ii*. *MPK-Gen* (generating the master key and public key); *iii*. *IK-Gen* (create the individual key through the *MK* and fingermarks); *iv*. *Sign* (use *IK* to generate a signature for signing the incoming data from nodes and setting up an access policy); and, *v*. *Verify* (use *PK*, signatures, and the access policy to verify the identification of the data transmitter). After all sensitive processes have been executed in TEE, the results will be sent back to REE for later use.

**Figure 5:**
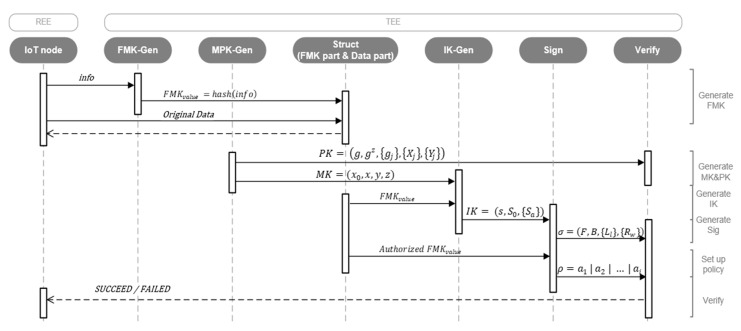
The sequence diagram of the architectural algorithm.

Figure 5 elaborates on the execution process by formulating several algorithms in detail. At the end of the process, the SNPL scheme can distinguish malicious nodes from secure ones.

#### 3.2.1. FMK-Gen

In this step, the device node’s unique authentication information is used to generate a safe value to be used later.

At first, the system defines a structure for each IoT device, which consists of two parts, FMKpart and Data part expressed as Struct={FMK part, Data part} Meanwhile, suppose that all IoT nodes with sensors are placed in REE. By the interaction process, IoT nodes transmit gathered data with their configuration information from REE to the *FMK-Gen* module in TEE. 

After that, the *FMK-Gen* module extracts the identity information, such as UUID and International Mobile Equipment Identity (IMEI), from equipment nodes, and then uses the hash algorithm to generate a secure unique value $FMK_{value}$. This process can be expressed as: $FMK_{value}=hash\left(info\right)$. Here the Message-Digest Algorithm 5 (MD5) from OpenSSL library is used to calculate MD5 values as $FMK_{value}$. 

At the end of this step, the system puts $FMK_{value}$ into FMKpart of Struct. Meanwhile, the original data is sent into Data part by IoT terminal nodes. This can be expressed as: $FMK\;part\;\leftarrow FMK_{value\;}\mathrm{and}\;Data\;part\leftarrow original\;Data$.

Now, each equipment node corresponds to a structure built based on its unique identity and gathered data. The process can be realized by Algorithm 1 shown below, where *i* represents the current node and sum represents the maximum number of nodes:
**Algorithm 1** Fingermark Generation**Input:** unique information of an IoT device info**Output:** IoT node’s $FMK_{value}$
**Define**Struct←{FMK part, Data part};**For**i←0**to***sum***do**$FMK_{value}\leftarrow hash\left(info\right)$;$FMK\;part\;\leftarrow FMK_{value}$;Data part←original Data;**End for****Return**$FMK_{value}$;

#### 3.2.2. MPK-Gen

In this step, the following groups and functions are defined first: two cyclic groups $Z_{p}^{*}$ and $Z_{p}$ in which prime order p is the size of the group, universe of attributes $U=Z_{p}^{*}$, a collision-resistant hash function $H:{\left\{0,1\right\}}^{*}\to Z_{p}^{*}$, and two cyclic groups $G_{1}$ and $G_{2}$ of size *p* that are equipped with a bilinear pairing $e:G_{1}\times G_{2}\to G_{T}$. Then, the required generators and parameters are emerged as follows:

(1) $$g\leftarrow G_{1};g_{0},\ldots ,g_{w_{max}}\leftarrow G_{2};x_{0},x,y,z\leftarrow Z_{p}^{*};$$

where g is a generator of $G_{1}$, $g_{0},\ldots ,g_{w_{max}}$ are *generators* of $G_{2}$, $x_{0},x,y,z$ are randomly chosen from $Z_{p}^{*}$, respectively. Then, we set the following values:

(2) X0=g0x0,Xj=gjx+z (∀j∈[0,wmax]); Yj=gj1y.

Next, the master key $MK=\left(x_{0},x,y,z\right)$ and the public key $PK=\;\left(g,g^{z},\left\{g_{j}|j\in \left[0,w_{max}\right]\right\},\left\{X_{j}|j\in \left[0,w_{max}\right]\right\},\left\{Y_{j}|j\in \left[1,w_{max}\right]\right\}\right)$ can be generated.

The process is summarized in Algorithm 2:
**Algorithm 2** Master Key and Public Key Generation**Input:** cyclic groups $Z_{p}^{*}$ and $Z_{p}$ of size *p*, cyclic groups $G_{1}$ and $G_{2}$ of size *p***Output:**MK and PK
**Define**$U\leftarrow \;Z_{p}^{*}$;$Z_{p}^{*}\leftarrow H:{\left\{0,1\right\}}^{*}$;$G_{T}\leftarrow e:G_{1}\times G_{2}$;**Choose**$g\leftarrow G_{1}$;$g_{0},\ldots ,g_{w_{max}}\leftarrow G_{2}$;$x_{0},x,y,z\leftarrow Z_{p}^{*}$;**For**j←0**to**$w_{max}$**do**$X_{0}\leftarrow g_{0}{}^{x_{0}}$;$X_{j}\leftarrow g_{j}{}^{x+z}$;Yj←gj1y; **End for**$MK\leftarrow \left(x_{0},x,y,z\right)$;$PK\leftarrow \left(g,g^{z},\left\{g_{j}|j\in \left[w\right]\right\},\left\{X_{j}|j\in \left[w\right]\right\},\left\{Y_{j}|j\in \left[1,w_{max}\right]\right\}\right)$;**Return**MK and PK;

#### 3.2.3. IK-Gen 

In this step, it is assumed that $U^{\prime }\subseteq U$; and $\forall a\in U^{\prime }$, where $U^{\prime }$ is an attribute set that contains attributes satisfying the access policy, and a is one of the legal attributes. For a randomly selected generator $s\leftarrow G_{1}$, the following values can be set:

(3) S0=s1x0z,Sa=s1(x+ya)

Note that $FMK_{value}$ is used to construct the value a, which is one of $a\in U^{\prime }$:

(4) $$a\leftarrow FMK_{value}$$

According to the generated MK and the attribute set $U^{\prime }$, the user’s individual key is:

(5) $$IK_{U^{\prime }}=\left(s,S_{0},\left\{S_{a}\right\}\right)$$

The above process can be summarized as Algorithm 3:
**Agorithm 3** Individual Key Generation**Input:** master key MK and Fingermark $FMK_{value}$ of an IoT node**Output:** individual key IK of an IoT node**Define**$U^{\prime }\subseteq U$;$\forall a\in U^{\prime }$;**Choose**$s\leftarrow G_{1}$;**For**i←0**to***sum***do**$a\leftarrow FMK_{value}$;S0←s1x0z;Sa←s1(x+ya);**End for**$IK_{U^{\prime }}\leftarrow \left(s,S_{0},\left\{S_{a}\right\}\right)$;**Return**$IK_{U^{\prime }}$;

#### 3.2.4. Sign

Firstly, an access policy is set up to decide which user can get into the system on the basis of its attributes. Then, the *Sign* module defines a predicate $\rho \left(U^{\prime }\right)=1$; and calculates a matrix $M_{l\times w}$ based on the predicate, and a label vector $a_{i}$, which indicates the relationship between an attribute and its corresponding row. This step means that policy ρ corresponds to the monotone span program $M\in {\left(Z_{p}\right)}^{l\times w}$ with the row labeling a:{l}→U. According to the program M given above, a vector v corresponding to $U^{\prime }$ is computed through the following rules: $v_{i}$ has two values of 0 and 1, which $v_{i}=1$ means the corresponding attribute $a_{i}$ is used in the access policy while $v_{i}=0$ means $a_{i}$ is not used or does not exist. Finally, ε is calculated as ε=H(d‖ρ). 

Note that only the set of accessible $FMK_{value}$ of authorized nodes is applied to the policy ρ. The $FMK_{value}$ of those inaccessible nodes are not included in ρ. The policy ρ is constructed through OR operation with authorized $FMK_{value}$ as follows:

(6) $$\rho =a_{1\;}\left|a_{2}\;\right|\;\ldots \;|\;a_{i}$$

where $a_{1}=FMK_{value1},a_{2}=FMK_{value2}\ldots a_{i}=FMK_{valuei}$. The process can be summarized in Algorithm 4:

Choose random generators $t_{0}$ from $Z_{p}^{*}$ and $t_{1},t_{2},\ldots t_{l}$ from $Z_{p}^{*}$, which are expressed as $t_{0}\leftarrow Z_{p}^{*}$ and $t_{1},t_{2},\ldots t_{l}\leftarrow Z_{p}$, respectively. Then, set values $F,\;B,L_{i}$ and $R_{j}$ where ∀i∈{l} and ∀j∈{w}:

(7) $$F=s^{t_{0}},B=S^{t_{0}+z}$$

(8) $$L_{i}={\left(S_{a_{i}}{}^{v_{i}}\right)}^{t_{0}}\cdot g^{t_{i}\left(z+\varepsilon \right)};R_{j}=\prod_{i=1}^{l}{\left(X_{j}Y_{j}{}^{a_{i}}\right)}^{M_{ij}\cdot t_{i}}$$

The signature for signing the data in *Datapart* is set as $\sigma =\left(F,B,L_{1}\ldots L_{l},R_{1}\ldots R_{w}\right)$. The process can be summarized in Algorithm 5.
**Algorithm 4** Set up An Access Policy**Input:**$FMK_{value}$ of addressable IoT nodes**Output:** an access policy ρ
**Define**$\rho \left(U^{\prime }\right)\leftarrow 1$;$M_{l\times w}\leftarrow M\in {\left(Z_{p}\right)}^{l\times w}$;a:{l}→U;ε←H(d‖ρ);**For**i←0**to**l**do**$a_{j}\leftarrow {\mathrm{FMK}}_{\mathrm{valuei}}$**End for**$\rho \leftarrow a_{1\;}\left|\;a_{2}\;\right|\;\ldots \;|\;a_{i}$;**Return** 0;
**Algorithm 5** Signature Generation**Input:** individual key IK of an IoT node**Output:** signature σ of a specific IoT node**Choose**$t_{0}\leftarrow Z_{p}^{*}$;$t_{1},t_{2},\ldots t_{l}\leftarrow Z_{p}$;**For**i←0**to**l**&&**j←0**to**w**do**$F\leftarrow s^{t_{0}}$;$B\leftarrow S_{0}{}^{t_{0}+z}$;$L_{i}\leftarrow {\left(S_{a_{i}}{}^{v_{i}}\right)}^{t_{0}}\cdot g^{t_{i}\left(z+\varepsilon \right)}$;$R_{j}\leftarrow \prod_{i=1}^{l}{\left(X_{j}Y_{j}{}^{a_{i}}\right)}^{M_{\mathrm{ij}}\cdot t_{i}}$;**End for**$\sigma \leftarrow \left(F,B,L_{1}\ldots L_{l},R_{1}\ldots R_{w}\right)$;**Return**σ;

#### 3.2.5. Verify

Based on PK, σ, and ρ generated in the above algorithms, the system can determine whether an input should be accepted or rejected. The result SUCCEED means the node gets the authority successfully, while the result FAILED means the node is access-denied. The first step is checking the value of F. F=1 indicates FAILED. For F=0, the following conditions need to be check for all j∈{w}:

1. $e\left(B,g_{0}{}^{x_{0}}\right)$ = e(sF1z,g0);
2. For j=1, ∏i=1le(Li,gj(x+aiy+z)·Mij) = e(F,g11z)e(g,R1z+ε); for j≠1, ∏i=1le(Li,gj(x+aiy+z)·Mij) = $e\left(g^{z+\varepsilon },R_{j}\right)$.

If all od the above conditions hold, the results is SUCCEED. The system then transmits the result from TEE to REE. The incoming data in Datapart of authorized nodes will be sent back to REE for further use. Meanwhile, the data from rejected nodes are deleted. This process can be summarized in Algorithm 6:
**Algorithm 6:** Verify**Input:** public key PK, signature σ and predefined access policy ρ
**Output:** SUCCEED or FAILED**If**F=1**Then** FAILED;**Else****Check**$e\left(B,g_{0}{}^{x_{0}}\right)$ = e(sF1z,g0);**If**YES**and**j=1**Check**∏i=1le(Li,gj(x+aiy+z)·Mij) = e(F,g11z)e(g,R1z+ε);**If**YES, **return** SUCCEED;**Else if**YES**and**j≠1**Check**∏i=1le(Li,gj(x+aiy+z)·Mij) = $e\left(g^{z+\varepsilon },R_{j}\right)$;**If**YES, **return** SUCCEED;**Else return** FAILED;

### 3.3. Security Proof

This section provides a security proof of our scheme, which covers illustrations of correctness, privacy, and unforgeability with formulations. The scheme satisfying the proof can be considered as a safe solution once proved correct, entirely private and unforgeable [20]. 

Correctness means that in the light of PK, MK, IK, access policy, and correspondingly generated true signatures, correct verification results and equations can be obtained in the process of verification. By the detailed explanation in Section 3.2 and the direct substitution method, it is obvious that the correctness is fulfilled.

Privacy means the attacker never receive attributes and IK of a legal node by the generated signature. Here we can see that even though there are different attribute sets leading to different IKs, any legal attribute sets resulting Verify(PK,d,ρ,σ)=1 have the identical distribution while calculating signatures under the same ρ. When an attacker generates a hoped-for signature without legal attributes, there is a neglectable possibility to gain a signature satisfying the access policy, which makes $\rho \left(U^{\prime }\right)=1$ and Verify(PK,d,ρ,σ)=1. Still, the terms in $\sigma =\left(F,B,L_{1}\ldots L_{l},R_{1}\ldots R_{w}\right)$ are unique corresponding while successfully verified. So, the IK and signature generated at different times are distinct, which draws to the conclusion that privacy is guaranteed.

The unforgeability means the success probability of an attacker in any polynomial times is ignorable when faced with the following circumstance:

1. Generate public key and master key by (*PK*, *MK*) ← *MPK-Gen*, and send the results to the attacker; and,
2. The attacker has access to the *IK-Gen* module and *Sign* module such that it can generate a forged signature $\sigma^{*}$ to pass the validation of the access policy.

In other words, when an attacker has incorrect access structure and inappropriate attributes, but eventually gains a correct verification result, we can say that the attacker succeeds to get access.

The remaining of this section verifies this property. Let $M\in {\left(Z_{p}\right)}^{l\times w}$ be the monotone span program of ρ, a be the row labeling that a:{l}→U, and ε=H(d‖ρ). Then, the following steps are implemented:

1. Randomly choose $h_{1},\ldots ,h_{l}\leftarrow Z_{p}$ and $u\leftarrow Z_{p}^{*}$;
2. Calculate $r_{j}=\frac{1}{y\left(z+\varepsilon \right)}\left\{\sum_{i=1}^{l}\frac{\left[\left(x+ya_{i}\right)h_{i}-uv_{i}\right]\left(xy+yz+a_{i}\right)M_{i,j}}{x+ya_{i}}\right\}$ for all j∈[w];
3. Let the signature $\sigma =\left(\sigma_{1},\sigma_{2},\sigma_{3},\sigma_{4}\right)$, where $\sigma_{1}=g^{t_{0}s}$, $\sigma_{2}=g^{s\left(t_{0}+z\right)/x_{0}z}$, $\sigma_{3}=\left\{g^{h_{i}}|i\in \left[l\right]\right\}$, $\sigma_{4}=\left\{g_{j}^{r_{j}}|j\in \left[w\right]\right\}$.

It is necessary to use the programmatic techniques of universal groups to certify the unforgeability as shown below. Before formal certification, parameters are set similar to previous algorithms in Section 3.2. An assumption is then made that the fake signature of an attacker is defined as: $\sigma^{*}=\left(g^{u^{*}},g^{b^{*}},\left\{g^{h_{i}^{*}}|i\in \left[l\right]\right\},\left\{g^{r_{j}^{*}}|j\in \left[w\right]\right\}\right)$, with data $d^{*}$ and access policy $\rho^{*}$, where $\left(d^{*},\rho^{*}\right)\neq \left(d^{\left(q\right)},\rho^{\left(q\right)}\right)$. Similarly, let $M^{*}\in {\left(Z_{p}\right)}^{l^{*}\times w^{*}}$, of which $a^{*}$ is the row labeling. At last, let $\varepsilon^{*}=H(d^{*}\|\rho^{*})$.

Note that $u^{*}\neq 0$ and $b^{*}=\frac{s\left(t_{0}+z\right)}{x_{0}z}$. To construct the counterfeit signature, the following equation is constructed:

(9) $$\sum_{i=1}^{l^{*}}h_{i}^{*}M_{i,j}^{*}\left(xy+yz+a_{i}^{*}\right)\Delta_{j}\;=\overset{l^{*}}{\underset{i=1}{\sum }}\frac{xy+yz+a_{i}^{*}}{x+ya_{i}^{*}}u^{*}{v^{\prime }}_{j}\Delta_{j}+y\left(z+\varepsilon^{*}\right)r_{j}^{*},j\in \left[w^{*}\right]$$

where ${v^{\prime }}_{j}=\left[1,0,\ldots ,0\right]$. Here a hypothesis is that the above equation holds, followed with getting a contradictory result. That is to say, here reduction to absurdity is used as an effective method, aiming at reaching an outcome that the attacker can produce a legitimate signature using IK, so that the signature is not a counterfeit.

Let Lin(P) be the collection of multilinear polynomials, which P is defined as:

(10) $$P=\left\{1,x_{0},\Delta_{0},z\right\}\;\cup \;\left\{\Delta_{j},\left(x+z\right)\Delta_{j},\Delta_{j}/y|j\in \left[w\right]\right\}\;\cup \;\left\{o_{s},o_{s}/x_{0}z,o_{s}/\left(x+ay\right)|o\in \left[n\right],a\in {U^{\prime }}_{s}\right\}\;\cup \;\left\{h_{i}^{\left(q\right)},u^{\left(q\right)},b^{\left(q\right)},r_{j}^{\left(q\right)}|q\in \left[v\right],i\in \left[l^{\left(q\right)}\right],j\in \left[w^{\left(q\right)}\right]\right\}$$

where $o\in Z_{p}^{*}$ is chosen randomly and P is the attribute set with coefficients in $Z_{p}^{*}$. Meanwhile, let Hom(P) be the collection of homogeneous polynomials, which is the subset of a multilinear polynomial set, i.e., Hom(P)⊂Lin(P). As our proof is based on the mathematical theory of multilinear functions and homogeneous polynomial, it comes to the conclusion that the expressions that are provided by the counterfeit of an attacker cannot embody certain specific terms.

Since it is obviously that $u^{*},b^{*},\left\{h_{i}^{*}|i\in \left[l\right]\right\},\left\{r_{j}^{*}|j\in \left[w\right]\right\}\in Lin\left(P\right)$, as well as $u^{*}=b^{*}\frac{x_{0}t_{0}z}{t_{0}+z}$, we can conclude that:

(11) $$u^{*}\in Hom\left(\left\{\Delta_{0}x_{0}\right\}\cup \;\left\{o_{s}|s\in \left[n\right]\right\}\cup \;\left\{u^{\left(q\right)}|q\in \left[v\right]\right\}\right)$$

Since $\Delta_{j}|y\left(z+\varepsilon^{*}\right)r_{j}^{*}$ and consequently $\Delta_{j}|r_{j}^{*}$, we get:

(12) $$r_{j}^{*}\in Hom\left(\left\{\Delta_{j},\Delta_{j}\left(x+z\right),\Delta_{j}/y\right\}\cup \;\left\{r_{j}^{\left(q\right)}|q\in \left[v\right]\right\}\right)$$

Here it is assumed that ${v^{\prime }}_{j_{0}}\neq 0$ and $u^{*}$ includes term $\Delta_{0}x_{0}$. Therefore, $\sum_{i=1}^{l^{*}}\frac{xy+yz+a_{i}^{*}}{x+ya_{i}^{*}}u^{*}{v^{\prime }}_{j}\Delta_{j_{0}}$ contains $\Delta_{0}x_{0}\Delta_{j_{0}}$. Note that $\Delta_{0}x_{0}\Delta_{j_{0}}$ is impossible to exist in $y\left(z+\varepsilon^{*}\right)r_{j_{0}}^{*}$, or appear in $\sum_{i}^{l^{*}}h_{i}^{*}M_{i,j_{0}}^{*}\left(xy+yz+a_{i}^{*}\right)\Delta_{j_{0}}$. As a result, it concludes that:

(13) $$u^{*}\in Hom\left(\left\{o_{s}|s\in \left[n\right]\right\}\cup^{\;}\left\{u^{\left(q\right)}|q\in \left[v\right]\right\}\right)$$

Assume that there is a term $\Delta_{j}$ in $r_{j}^{*}$. As $u^{*}$ has no constant term, leading that $u^{*}{v^{\prime }}_{j}\Delta_{j}$ is incapable of contributing $\Delta_{j}$ to the equation. It is the same as $\sum_{i=1}^{l^{*}}h_{i}^{*}M_{i,j}^{*}\left(xy+yz+a_{i}^{*}\right)\Delta_{j}$. But it is necessary to contribute $\Delta_{j}$ and $\Delta_{j}/y$ for the equation’s right side, so:

(14) $$r_{j}^{*}\in Hom\left(\left\{\Delta_{j}(x+z),\Delta_{j}/y\right\}\cup^{\;}\left\{r_{j}^{\left(q\right)}|q\in \left[v\right]\right\}\right)$$

Assume that $r_{j}^{*}$ has the term $r_{j}^{\left(q\right)}$, it is necessary to bring $y\left(z+\varepsilon^{*}\right)r_{j}^{*}$ to the equation’s right side, which generates a term with the coefficient of $y\left(z+\varepsilon^{*}\right)/y\left(z+\varepsilon^{\left(q\right)}\right)$. $u^{*}$ and $\left\{h_{i}^{*}|i\in \left[l\right]\right\}$ cannot contribute $y\left(z+\varepsilon^{*}\right)/y\left(z+\varepsilon^{\left(q\right)}\right)$ to the equation As $\varepsilon^{*}$ and $\varepsilon^{\left(q\right)}$ are always different, therefore, we can conclude:

(15) $$r_{j}^{*}\in Hom\left(\left\{\Delta_{j}\left(x+z\right),\Delta_{j}/y\right\}\right)$$

As mentioned early, it is assumed that ${v^{\prime }}_{j_{0}}\neq 0$ for $j_{0}$. As $y\left(z+\varepsilon^{*}\right)r_{j_{0}}^{*}$ and $\sum_{i}^{l^{*}}h_{i}^{*}M_{i,j_{0}}^{*}\left(xy+yz+a_{i}^{*}\right)\Delta_{j_{0}}$ cannot provide the term $u^{\left(q\right)}$, $u^{*}$ is impossible to contain the monomial $u^{\left(q\right)}$. Accordingly,

(16) $$u^{*}\in Hom(\left\{o_{s}|s\in \left[n\right]\right\}\})$$

Ultimately, we can come to a conclusion that:

(17) $$r_{j}^{*}\in Hom\left(\left\{\Delta_{j}\left(x+z\right),\Delta_{j}/y\right\}\right)$$

(18) $$u^{*}\in Hom(\left\{o_{s}|s\in \left[n\right]\right\}\})$$

To make the Equation (9) tenable, let $h_{i}^{*}=w_{i}^{*}\left(O_{i}\right)+\delta^{*}\left(R/O_{i}\right)$ where $O_{i}=\left\{o_{s}/x+a_{i}y|a_{i}\in {U^{\prime }}_{s},s\in \left[n\right]\right\}$, for any terms of $u^{*}$ should also be provided by the left side of (9) to realize the equality of this expression. Here we divide $h_{i}^{*}$ into two addends to actualize the situation that $o_{k}$ only exists in one part of $h_{i}^{*}$. There is:

(19) $$\sum_{i=1}^{l^{*}}w_{i}^{*}M_{i,j}^{*}\left(xy+yz+a_{i}^{*}\right)=\sum_{i}^{l^{*}}\frac{xy+yz+a_{i}^{*}}{x+{ya}_{i}^{*}}u^{*}{v^{\prime }}_{j}\left(j\in \left[w\right]\right)$$

Here a vector $v_{i}^{*}$ is defined, which makes $v^{*}M^{*}=v^{\prime }{}_{1}\ldots v^{\prime }{}_{t}=1,0,\ldots ,0$ hold. Besides, according to prior works, $U^{\prime }{}_{s_{0}}$ should comprise the attribute $a_{i}^{*}$. Then, $v_{i}^{*}$ can be constructed as follows:

(20) $$v_{i}^{*}=\left[\frac{o_{s_{0}}}{x+ya_{i}^{*}}\right]w_{i}^{*}/\left[o_{s_{0}}\right]\left(i\in \left[l\right]\right)$$

in which $\left[o_{s_{0}}\right]$ is the coefficient of $o_{s_{0}}$ in $u^{*}$ ($o_{s_{0}}\neq 0$). Based on the above mathematical and derivation, we have $\rho^{*}\left({U^{\prime }}_{s_{0}}\right)=1$, which confirms that the signature is not a counterfeit. That is to say, the property of unforgeability is proved.

## 4. Experimental Evaluation

In this section, details about experiments on simulating the SNPL framework are described. Besides, several performance measurements are defined such that the results can be analyzed and compared from several different aspects.

### 4.1. Experiment Design

**Figure 6:**
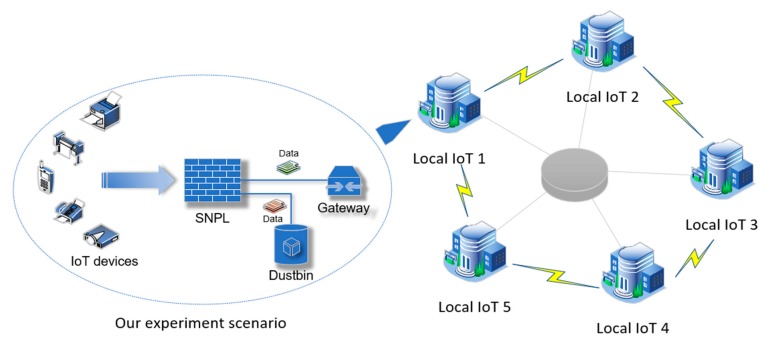
Scenario explanations of experiments and realistic scenes.

It is necessary to explain why experiments are not carried out on a large scale here. For the whole application scenario, the scheme simulated in experiments is a representative or an epitome of different local IoT, which is connected or communicating with each other. That is to say, other application scenarios are extensions of similar situations. So, conducting experiments in more extensive or more situations makes no sense, for those are seen as repetitive actions. The scenario explanation is shown in Figure 6.

Two experiments are conducted to simulate real IoT application scenarios. The first experiment is to test the effectiveness and verification capability of the SNPL scheme with a single node of different types. This experiment provides information on the success rate and means the processing time of one single node. The second experiment aims to evaluate the performance under different numbers of nodes. The goal is to test whether the number of devices has an impact on performance. In the experiments, the processing time of various nodes is recorded to calculate the mean value. Besides, the accuracy of different nodes is also recorded and compared to ensure the universality of the SNPL scheme.

**Figure 7:**
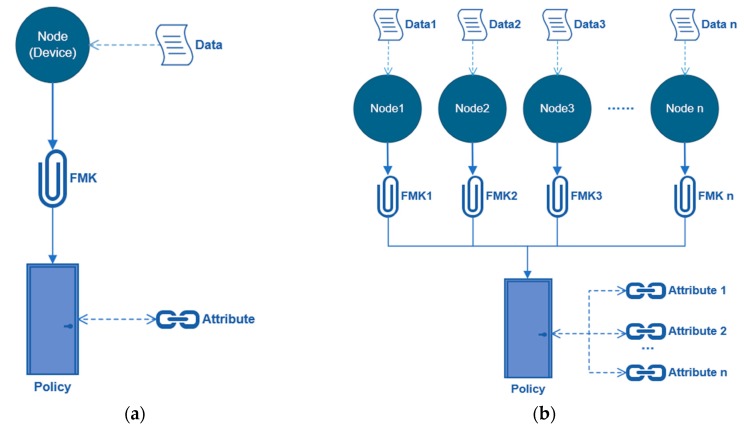
(**a**) The first test based on a single node; and, (**b**) The second test based on multiple nodes.

As mentioned early, the first experiment aims to evaluate the effectiveness of the SNPL scheme with one single node, as shown in Figure 7a. In this experiment, 20 different types of sensors are connected to the Raspberry PI and each sensor is a separate node. Our purpose is to test whether the SNPL scheme can identify the data source. The finger marks are generated by using the configuration text files of devices, which are stored in a specific folder. A policy with one legal attribute is then defined to verify nodes’ identification one by one. Among all of these nodes to be tested, the normal one represents the objective whose attribute is legal and set into the policy, while the malicious one represents the node whose attribute is illegal and not in the policy. Then, individual keys of devices are generated by using the fingermarks as an attribute in the algorithm. These individual keys are used to sign messages, after which the policy is used to provide verification of these nodes. In this process, the processing time of the SNPL scheme of 20 different nodes are recorded, their mean value is calculated, and the success rate is obtained. 

The second experiment is to compare the running time and accuracy of the algorithm under different quantities of nodes, as shown in Figure 7b. This experiment simulates a real-world scenario and evaluates whether the number of parallel nodes has an influence on the SNPL scheme. The operation is substantially similar to that of the first experiment, in which the only difference is the number of processed nodes. Here virtual nodes are defined with respective fingermarks for all targets and an access policy with all legal attributes (i.e., the attributes which satisfy the policy). Then, each defined node signs up one message and generates a signature for verifying with the policy. After that, the verification results are obtained. In this process, the processing time with a different number of nodes is recorded and compared. The accuracy in terms of throughput rate and blocking rate is also compared.

**Figure 8:**
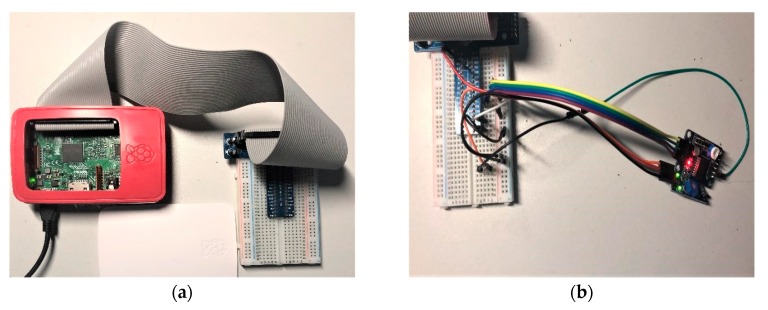
(**a**) Hardware device as an IoT node; and, (**b**) Breadboard with sensors installed.

The experiment is carried out on the Ubuntu operating system. To implement the proposed SNPL scheme, Raspberry Pi 3 with different types of sensors is used to simulate different IoT nodes (i.e., a temperature sensor attached to the Raspberry Pi 3 simulates the first node, while the photoelectric sensor attached can be the second simulated node, and others). Open-TEE is used to construct the TEE in Ubuntu, while the normal execution environment of Ubuntu is considered as the REE. The Ubuntu system is deployed in VMware workstation 14, which is installed on a Dell Inspiron 15-5577 notebook. Our platform is developed in the C language using qtcreator-3.6.1. The hardware are shown in Figure 8 and the device specifications are listed below:

Operating system: Ubuntu 14.04 LTS.
Hardware simulation: Raspberry Pi 3 with sensors.
CPU: Intel^®^ Core™ i7-7700HQ CPU @ 2.80GHz
Memory: 979.8 MB
Storage: 41.1 GBxc

The SNPL scheme can be taken as a “small nodes gateway” to control data transfer to the real gateway. The “small nodes gateway” can be replicated to simulate different scales of IoT. Therefore, the experiment can be seen as a classic case of different kinds of IoT environments and the conclusions can be extended to various real-world scenarios.

### 4.2. Experimental Results

In this section, experimental evaluation and comparison of the SNPL scheme are presented. First, the running time of the entire process of the experiments is discussed. Then, the accuracy under different types and numbers of nodes are compared.

#### 4.2.1. Processing Time

**Figure 9:**
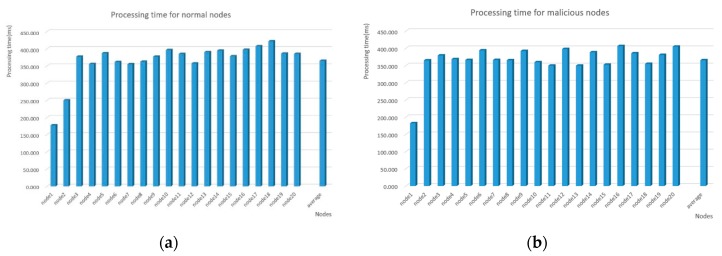
(**a**) Processing time for normal nodes; and, (**b**) Processing time for malicious nodes.

Figure 9a shows the processing time with 20 kinds of normal nodes. The result shows that the execution time varies with different nodes. In our experiments, the average execution time of 20 normal nodes is about 365.661 ms. Figure 9b shows the result of identifying 20 kinds of malicious nodes. Also, the SNPL scheme has different processing periods when dealing with different nodes. It must be said that the obvious discrepancy of node 1 is just due to its node type. And the difference of node 2 between normal and malicious properties is just an experimental error, which may be caused by the system operation time, the network latency, or other factors. The chart shows that the average time is approximately 365.676 ms. It can be concluded from the statistics that the processing time for the detection of normal and malicious nodes is comparable. In other words, the performance of the scheme on identifying nodes is not affected by the properties of nodes.

Table 1 shows the processing time with different numbers of nodes (1 node, 5 nodes, 10 nodes, 15 nodes, and 20 nodes) excerpted from the complete experimental results. In each case, the amounts of normal nodes and malicious nodes are shown to evaluate the verification accuracy.

**Figure 10:**
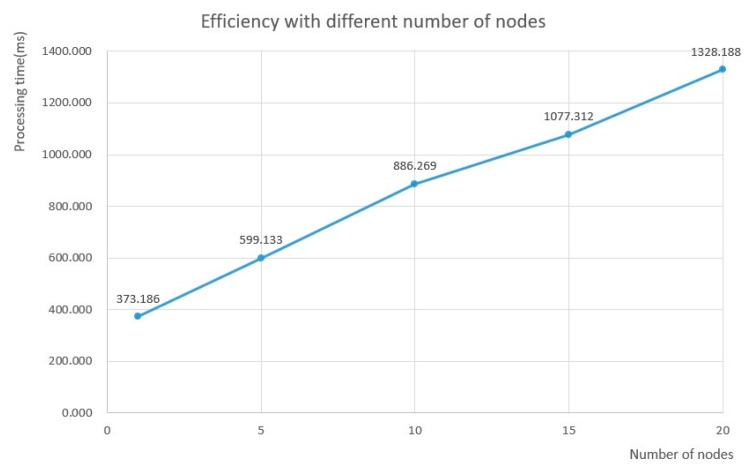
Efficiency changes in disposing of different numbers of nodes.

Figure 10 shows a line chart, given by efficiency vs. different number nodes, for an intuitive description of the results. Here the efficiency is represented by the processing time against different numbers of nodes. Shorter processing time indicates a higher efficiency of the SNPL scheme. The chart shows that the processing time increases almost linearly with the increase on the number of nodes. That is, there is a linear increase relationship between the number of nodes and processing time.

It must be stated that the scenario of a small-scale local IoT (i.e., a house, an office, or anywhere) does not contain a large number of smart nodes. To explain the performance results, here consider an application scenario in which a man wears some wearable devices. Within this scenario, the total amount of smart nodes the man carries will not usually exceed 10. That is, the quantity of nodes in a real application is generally within the validation range of our experiment. Based on experimental outcomes of total running time and identifying precision shown in this experiment, it can be concluded that our proposed scheme improves security without compromising performance.

#### 4.2.2. Accuracy

The first experiment shows that the SNPL scheme can recognize the identity of an inspected node correctly. During the experiment, all normal nodes are proved to be safe nodes as expected while all malicious nodes are proved unsafe. The results are obtained based on the following calculation expressions:

(21) $$AC_{sgl\_s}=\frac{T_{safe}}{N_{sum\_s}}AC_{sgl\_m}=\frac{T_{mali}}{N_{sum\_m}}$$

(22) $$AC_{sgl\_m}=\frac{T_{mali}}{N_{sum\_m}};$$

where $AC_{sgl\_s}$ and $AC_{sgl\_m}$ represent the accuracy of safe node detection and malicious node detection, respectively, $T_{safe}$ and $T_{mali}$ are the times of successful validations of safe and malicious nodes, respectively, $N_{sum\_s}$ and $N_{sum\_m}$ are the total number of safe and malicious nodes to be measured, respectively. The verification accuracy of single node identification is shown in Table 2.

In the second experiment, the accuracy is indicated by the percentage of all nodes that are verified correctly over the total number of nodes. Ideally, all nodes are verified correctly, regardless of the number of unidentified nodes. That is to say, on the premise that the total number of nodes unchanged, the amounts of normal nodes and malicious nodes do not affect the accuracy of recognition. The accuracy of detection on multi-node denoted as $AC_{mul}$, is given as:

(23) $$AC_{mul}=P_{rec\_suc}\;=\frac{N_{suc\_rec}}{N_{sum}}=\frac{N_{rec\_s}+N_{rec\_m}}{N_{sum}}$$

where $N_{rec\_s}$ and $N_{rec\_m}$ are the amounts of safe and malicious nodes distinguished successfully, respectively, while $N_{sum}$ is the total amounts of nodes under test.Experimental results indicate that that system accuracy is 100% no matter how the number of nodes changes. Some experimental results are tabulated in Table 3. In all cases, the accuracy of node authentication is 100% with a relatively short checking period. The results demonstrate the exceptional performance of SNPL scheme in identification.

## 5. Application

This section discusses three potential application scenarios for the proposed SNPL scheme.

### 5.1. Smart Healthcare

IoT technologies can benefit the healthcare domain, in which tracking, identification, and data collection are the most critical applications [24]. In this field, light-weight monitoring or wearable devices play essential roles in monitoring patients’ conditions, recording and sending their real-time data to doctors or hospitals automatically. These monitoring and wearable devices have access to a patient’s sensitive and private data such as blood pressure and heart rate, from different nodes. Assume that there exists a malicious node and this node can modify real data and send the revised information to hospitals or doctors, which may lead to misdiagnosis. In this situation, our security scheme can identify the malicious node and prevent the modification of critical data. In other words, SNPL can protect data trustworthily.

### 5.2. Smart Building

In IoT, many kinds of smart devices are used in a building, constituting a local sensor network. Sensors and actuators arranged in the building can make people’s life more comfortable. For example, rooms heating can adapt to the weather condition and our preferences; rooms lighting can change based on the time of a day; the electrical equipment can be automatically on/off to save energy; monitoring and alarm systems can avoid domestic incidents [25]. In a word, intelligent nodes with sensors keep watching on the status of the whole building. For better management and safety, a gateway node can be used for each family or office. All data collected by the smart equipment will be transferred and stored in the gateway node. To verify the security of the data, our SNPL security scheme can be used in the transmission between endpoint nodes and the gateway node, which can take precautions against forged nodes and invaders. Moreover, it is also useful in the protection of household information and office data.

### 5.3. Smart Transportation

Smart transportation, which is also known as intelligent transportation, is a typical IoT-based application [26]. This system consists of a high number of smart vehicles connected via wireless networks [27], and smart vehicles can perceive road conditions and share traffic information with others. For such, every vehicle is equipped with Electronic Control Units (ECUs) for controlling subsystems and sharing gathered data within the vehicle. Besides, vehicles can connect to external networks for communications [28]. However, as adversaries may take over ECUs through launching attacks against endpoint nodes and subsystems, through there are some data protection schemes [29], data can be modified and transferred to other ECUs or vehicles and cause damages to the whole transportation system [30,31]. To protect against the security thread, our SNPL security scheme can be deployed between subsystems and the ECU for a reliability check on source nodes’ identities and messages. As a result, malicious data from unsafe nodes do not get into the transportation network and the stability of the whole system can be guaranteed.

## 6. Conclusions

In this paper, a SNPL scheme is proposed to ensure the trustworthiness of IoT nodes that are based on nodes attributes. In such scheme, the MD5 algorithm is used to generate a private value for an IoT node that is used as an identification attribute of the IoT node. Next, the node’s attribute and predefined access policy are applied for node authentication in TEE, which is a trusted development circumstance for realizing sensitive operations. To demonstrate the effectiveness of the proposed scheme, a series of experiments are conducted to evaluate the performance in terms of processing time, accuracy and success rate. Experimental results show that the proposed SNPL security scheme can identify normal nodes and malicious nodes that are based on their unique identify information with high efficiency and accuracy. Also, this indicates that the proposed SNPL scheme can protect the security of the source data at the beginning of the IoT interaction flow. Future directions of this research include the investigation of optimized algorithms design and their implementation in real applications.

## Figures and Tables

**Table 1:** Processing time with different numbers of nodes.

Total Number of Emulated Nodes	Number of Normal Nodes	Number of Malicious Nodes	Processing Time(ms)
1	0	1	362.081
	1	0	384.290
5	0	5	744.768
	1	4	549.926
	2	3	564.753
	3	2	436.345
	4	1	680.267
10	0	10	908.323
	2	8	883.820
	4	6	878.198
	6	4	906.796
	8	2	953.472
15	0	15	1098.231
	3	12	1083.484
	6	9	1108.137
	9	6	1078.030
	12	3	1089.419
20	0	20	1318.808
	5	15	1463.790
	10	10	1449.573
	15	5	1125.775
	20	0	1278.245

**Table 2:** Accuracy result with a single node.

Secure Node			Malicious Node		
$N_{sum\_s}$	$T_{safe}$	$AC_{sgl\_s}$	$N_{sum\_m}$	$T_{mali}$	$AC_{sgl\_m}$
1	1	100%	1	1	100%
5	5	100%	5	5	100%
10	10	100%	10	10	100%
15	15	100%	15	15	100%
20	20	100%	20	20	100%

**Table 3:** Accuracy result with multiple nodes.

$N_{sum}$	$N_{rec\_s}$	$N_{rec\_m}$	$AC_{mul}$
5	0	5	100%
	1	4	
	2	3	
	3	2	
	4	1	
10	0	10	100%
	2	8	
	4	6	
	6	4	
	8	2	
15	0	15	100%
	3	12	
	6	9	
	9	6	
	12	3	
20	0	20	100%
	5	15	
	10	10	
	15	5	
	20	0	
